# Yuk-Gunja-Tang attenuates neuronal death and memory impairment *via* ERK/CREB/BDNF signaling in the hippocampi of experimental Alzheimer’s disease model

**DOI:** 10.3389/fphar.2022.1014840

**Published:** 2022-10-26

**Authors:** Malk Eun Pak, Hye Jin Yang, Wei Li, Jae Kwang Kim, Younghoon Go

**Affiliations:** Korean Medicine (KM)-Application Center, Korea Institute of Oriental Medicine, Daegu, South Korea

**Keywords:** cell death, cognition, hippocampus, ERK/CREB/BDNF signaling, alzheimer’s disease, yuk-gunja-tang

## Abstract

Yuk-Gunja-Tang (YG) is the Korean traditional medicine in East Asia for gastrointestinal disorders. In the present study, we determined the protective effects of YG on glutamate-induced cytotoxicity in HT22 hippocampal neuronal cells and mice with scopolamine-induced memory impairment. *In vitro* assessments were performed using a cell viability assay, flow cytometry, and Western blotting, while *in vivo* assessments were performed in C57BL/6 mice administered with YG for 7 days and injected with scopolamine (1 mg/kg) for 7 days. We assessed the memory function using the Y-maze, novel object recognition, and passive avoidance tests. Protein expression analyses and histological analyses were performed using hippocampal tissues. YG treatment significantly restored cell viability against glutamate-induced apoptosis. It significantly suppressed glutamate-induced reactive oxygen species accumulation and mitochondrial dysfunction. It also increased Bcl-2 protein expression and decreased HO-1 protein expression. It activated the extracellular signal-regulated kinase/cAMP response element binding protein (ERK/CREB) signaling pathway and increased the expression of brain-derived neurotrophic factor (BDNF) under excitotoxic conditions. In the scopolamine-injected mice, YG ameliorated memory impairment in the Y-maze, novel object recognition, and passive avoidance tests; restored dysfunction in the acetylcholine, acetylcholinesterase expression levels; reduced neuronal damage in Nissl staining; and increased BDNF and phosphorylated ERK and CREB levels in Western blotting and immunofluorescence staining. Thus, YG exerted neuroprotective effects by activating ERK/CREB/BDNF signaling in the hippocampus, indicating its potential cognition-enhancing effects, especially in Alzheimer’s disease.

## 1 Introduction

Alzheimer’s disease (AD) is one of the most common neurodegenerative disorders, and its primary clinical feature is progressive memory impairment (Selkoe, 2002). Although the exact causes of AD are unknown, its pathogenesis is associated with pathways of neuronal death, including oxidative stress-induced damage and dysfunction of cholinergic signaling (Guan, 2008; Ballinger et al., 2016). Neurotransmitters such as glutamate and acetylcholine (ACh) modulate cholinergic signaling, which is involved in memory and learning (O'Reilly and Rudy 2001; Giovannini et al., 2001). The cholinergic neurons project to the hippocampus; in AD, severe loss of these neurons contributes to memory impairment (Ferreira-Vieira et al., 2016).

Scopolamine is an ACh antagonist that causes cognitive decline by binding to the muscarinic receptors and blocking the cholinergic system. It has been used as an inducer of an experimental AD animal model (Klinkenberg and Blokland 2010). Glutamate is also involved in maintaining the normal brain function through regulation of synaptogenesis and neuronal survival; thus, it contributes to the memory process (Onaolapo et al., 2016). Excessive extracellular glutamate in the hippocampus induces cell death in a phenomenon called excitotoxicity (Kritis et al., 2015); it is also a part of the AD pathogenic cascade (Hynd et al., 2004).

Mitogen-activated protein kinases (MAPKs) participate in important signaling pathways that are involved in the regulation of cell proliferation, differentiation, and apoptosis. Extracellular signal-regulated kinase (ERK), a member of the MAPK family, regulates cell survival (Johnson and Lapadat 2002; Stathopoulou et al., 2008). cAMP response element-binding protein (CREB) is a transcription factor that regulates the brain-derived neurotrophic factor (BDNF) genes, and is associated with neuronal survival and synaptic plasticity. Activation of the ERK/CREB pathway increases BDNF production in the cortical neurons (Jeon et al., 2011), and BDNF protects the hippocampal neurons from glutamate toxicity through transient activation of ERK (Almeida et al., 2005).

Yuk-Gunja-Tang (YG), also known as Liu-Jun-Zi-Tang in China and Rikkunshito in Japan, is a traditional medicine in East Asia. It is composed of eight botanical drugs and has been used to treat upper gastrointestinal dysfunction (Saegusa et al., 2015). A previous study revealed that treatment with YG promotes gastric emptying (Tominaga et al., 2011) and ameliorates functional dyspepsia (Ko et al., 2018; Wang et al., 2018). However, the effects of YG on cognitive decline, protection of the hippocampal neurons, and reduction of neuronal death in pathological conditions (such as AD) have not yet been reported.

We hypothesized that YG administration would alleviate memory loss and neuronal death in neuropathological conditions. Therefore, we investigated the neuroprotective effects of YG against glutamate-induced cytotoxicity in HT22 hippocampal neuronal cells and against scopolamine-induced memory impairment in a mice model.

## 2 Materials and methods

### 2.1 Materials and chemicals

Scopolamine, donepezil, L-glutamine and glutamate, methylthiazole tetrazolium (MTT), DiOC_6_(3) were purchased from Sigma-Aldrich (St. Louis, MO, United States). 5-(and-6)-carboxy-2′,7′-dichlorodihydrofluorescein diacetate (carboxy-H_2_DCFDA) was purchased from Sigma-Aldrich (St. Louis, MO, United States) and Invitrogen (Thermo Fisher Scientific; Pittsburgh, PA, United States). Fetal bovine serum (FBS), Dulbecco’s modified Eagle’s medium (DMEM), and bovine serum albumin (BSA) were purchased from HyClone (Logan, UT, United States). Penicillin/streptomycin was purchased from Gibco (Thermo Fisher Scientific; Carlsbad, CA, United States), while Cell Counting Kit-8 (CCK-8) was purchased from Dojindo Molecular Technologies (Kumamoto, Japan). The FITC Annexin V/propidium iodide (PI) apoptosis detection kit was purchased from BD Biosciences (San Diego, CA, United States). Various primary antibodies were acquired from Cell Signaling Technology (Danvers, MA, United States), Santa Cruz Biotechnology (Dallas, TX, United States), and Novus Biologicals (Minneapolis, MN, United States). Horseradish peroxidase (HRP)-conjugated secondary antibodies were purchased from Bethyl Laboratories (Farmingdale, NY, United States). A polyvinylidene fluoride (PVDF) membrane was purchased from Whatman (Piscataway, NJ, United States) and an enhanced chemiluminescence (ECL) solution was purchased from Pierce (Rockford, IL, United States).

Thermo Dionex UltiMate 3000 (Thermo Fisher Scientific, San Jose, CA, United States) was used for high-performance liquid chromatography (HPLC); it is equipped with a binary pump, an auto-sampler, a column oven, a diode array UV/VIS detector, and a charged aerosol detector (CAD). HPLC-grade acetonitrile and methanol were obtained from Merck KGaA (Darmstadt, Germany). ACS reagent-grade formic acid was purchased from Sigma-Aldrich (St. Louis, MO, United States). For HPLC analysis, ultrapure water (18.2 MΩ cm^−1^) was obtained by purification with a Puris-Evo UP Water system equipped with Evo-UP Dio VFT and Evo-ROP Dico20 (Mirae ST Co., Ltd., Anyang, Gyeonggi-do, Korea). The reference standards used for HPLC analysis, including 6-gingerol, liquiritin, and liquiritigenin, were purchased from Sigma Aldrich (St. Louis, MO, United States). Hesperidin was purchased from Supelco (Belle-fonte, PA, United States). Atractylenolide I, atractylenolide III, ginsenoside Rb1, and ginsenoside Rg1 were obtained ChemFaces (Wuhan, China). Glycyrrhizin and pachymic acid were purchased from PhytoLab GmbH & Co. (Vestenbergsgreuth, Germany). The purity of all reference standards was above 95%.

### 2.2 Preparation of YG

The composition of YG is shown in Table 1. The YG powder was purchased from Hankook Sinyak Corp. (Chungnam, Korea). The YG powder (No. 18562) comprised *Panax ginseng* C.A. Mey [Araliaceae; Gingseng Radix], *Atractylodes macrocephal* Koidz. [Asteraceae; Atractylodis Rhizoma], *Poria cocos* Wolf [Polyporaceae; Hoelen], *Pinellia ternata* (Thunb.) Makino [Araceae; Pinelliae Rhizoma], *Citrus × aurantium* f. *deliciosa* (Ten.) M.Hiroe [Rutaceae; Aurantii Nobilis Pericarpium], *Glycyrrhiza uralensis* Fisch [Fabaceae; Glycyrrhizae Radix], *Zingiber officinale* Roscoe [Zingiberaceae; Zingiberis Rhizoma], and *Ziziphus jujuba* Mill [Rhamnaceae; Zizyphi Fructus] and was as water extract (yield: 15%). This powder was dissolved in water for use in all experiments.

**TABLE 1:** The Complete species and drug name of Yuk-gunja-tang (YG) and it is the composition ratio.

Drug name	Family	Scientific name	Parts	Composition ratio (per 100 g)
Gingseng Radix	Araliaceae	*Panax ginseng* C.A. Mey	Root	15.66
Atractylodis Rhizoma alba	Asteraceae	*Atractylodes macrocephal* Koidz	Root and rhizome	15.66
Hoelen	Polyporaceae	*Poria cocos* Wolf	Sclerotium	15.66
Pinelliae Rhizoma	Araceae	*Pinellia ternata* (Thunb.) Makino	Tuber	15.66
Aurantii Nobilis Pericarpium	Rutaceae	*Citrus × aurantium* f. *deliciosa* (Ten.) M.Hiroe	Fruit peel	15.66
Glycyrrhizae Radix	Fabaceae	*Glycyrrhiza uralensis* Fisch. Ex DC.	Root and rhizome	5.90
Zingiberis Rhizoma	Zingiberaceae	*Zingiber officinale* Roscoe	Rhizome	7.90
Zizyphi Fructus	Rhamnaceae	*Ziziphus jujuba* Mill	Fruit	7.90

### 2.3 HPLC-CAD analysis

HPLC analysis was performed using an HPLC system equipped with CAD to identify the phytochemicals of YG (see Supplementary Material). The chromatographic separation was carried out using a XBridge^®^ C18 column (4.6 × 150 mm, 5 μm; Waters Corp., Milford, MA, United States) with an XBridge^®^ BEH C18 VanGuard cartridge (3.9 × 5 mm, 5 μm; Waters Corp.). The temperature of the column oven was maintained at 40°C, and the injection volume for each sample was 10 µL. The mobile phase consisted of 0.1% formic acid (v/v) in water (A) and acetonitrile (B). To improve the chromatographic separation capacity, the gradient elution system was programmed at a flow rate of 1.0 ml/min as follows: 5% B, 0–5 min; 5%–10% B, 5–10 min; 10%–35% B, 10–30 min; 35%–45% B, 30–40 min; 45%–80% B, 40–55 min; 80% B, 55–65 min; 80%–100% B, 65–70 min; 100% B, 70–80 min; 100%–5% B, 80–81 min; and 5% B, 81–90 min. The CAD channel was optimized using the following parameters: power function, 1.00; data collection rate, 10 Hz; filter, 0.1; peak width, 0.02 min; control evaporator temperature, low; and wait ready, ± 5.0 K. All data acquisition and analyses were performed using the Dionex Chromelon software (Thermo Fisher Scientific, Waltham, MA, United States).

### 2.4 Cell culture

The mouse hippocampus-derived cell line, HT22, was cultured in DMEM supplemented with 10% FBS and 1% penicillin/streptomycin; the cells were incubated at 37°C in 5% CO_2_. The cells were then cultured at a density of 3 × 10^3^ cells/well in a 96-well plate or 2 × 10^4^ cells/well in a 6-well plate; these plates were then incubated for 24 h. After incubation with various concentrations of YG for 24 h, the cells were further incubated under the absence/presence of 5 mM glutamate for 24 h.

### 2.5 Cell viability assay

The MTT assay was used for determination of cell viability. After treatment, 10% MTT solution (5 mg/ml) was added into the plate. Following incubation in the dark for 2 h at 37°C, the absorbance of the samples was determined at 540 nm using a microplate reader (SpectraMax i3x, Molecular devices, Sunnyvale, CA, United States). The results are expressed as a percentage of the control cells.

### 2.6 Cell death assessment by flow cytometry

For determination of apoptotic cell death and the mitochondria membrane potential, a flow cytometric analysis was performed using the FITC Annexin V/PI apoptosis detection kit and DiOC_6_(3). The cells were harvested and washed with Phosphate-Buffered Saline (PBS). Each sample was treated with 5% Annexin V and 5% PI or a 10 nM DiOC_6_(3) solution and incubated for 20 min. The samples were analyzed using a CytoFLEX Flow Cytometer (Beckman Coulter, Brea, CA, United States).

### 2.7 Measurement of reactive oxygen species

For determination of oxidative stress, a flow cytometric analysis was performed using carboxy-H_2_DCFDA. After treatment, the harvested cells were washed with PBS and incubated with 20 μM carboxy-H_2_DCFDA for 60 min in the darkness. The samples were then analyzed using a CytoFLEX Flow Cytometer (Beckman Coulter, Brea, CA, United States).

### 2.8 Scopolamine-induced cognitive deficit mouse model

**FIGURE 1:**
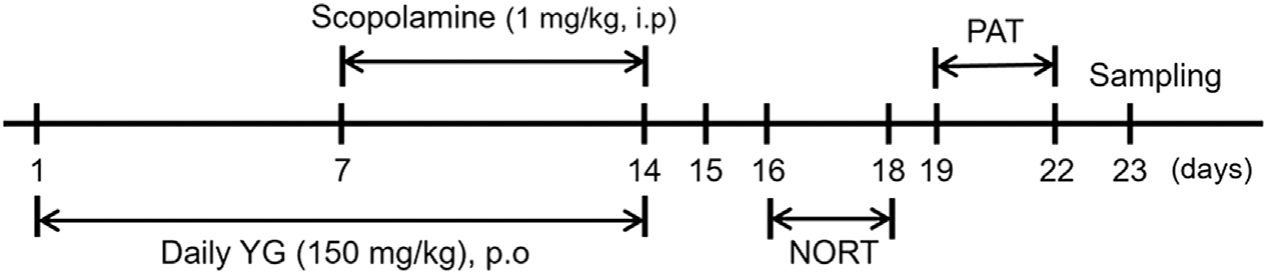
Schedule of the overall *in vivo* experiment.

Five-week-old male C57BL/6J mice (n = 21) were purchased from DooYeol Biotech (Seoul, South Korea); the mice weighed 16–18 g. All experiments were performed in accordance with the NIH guidelines for the care and use of laboratory animals; these experiments were approved by the Institute of Animal Care and Use Committee of the Korea Institute of Oriental Medicine (approval number: KIOM-D-21-009). The mice were housed at constant temperature (21 ± 2°C) and humidity under a 12 h light/dark cycle after purchase. The 21 mice were divided into the following three groups: CON (orally administered with water), SCO (orally administered with water), and YG (orally administered with 150 mg/kg of YG). The effective YG dose was found to be 7.5 g for humans (∼60 kg) in a clinical trial (Suzuki et al., 2014), thus, we divided 7500 mg into 60 kg and the administration dose of YG for mice was set at 150 mg/kg (7.5 g/60 kg). The mice in the SCO and YG groups were intraperitoneally injected with scopolamine (1 mg/kg) to induce memory impairment; however, the mice in the CON group were injected with PBS after daily YG administration. The experimental schedule is shown in Figure 1.

### 2.9 Behavior test

#### 2.9.1 Y-maze

A Y-maze with three arms (placed at a 120° angle from each other) was used to assess the spatial reference memory of the mice (Kraeuter et al., 2019). The mice were trained for 15 min and tested for 5 min; with the total protocol time was 1 day. Briefly, for training, the mice were placed into the Y-maze with one arm closed off; they were allowed to explore the maze for 15 min. After 1 h, the mice were allowed to explore the three opened arms of the Y-maze for 5 min; the time spent in the newly opened arm (novel arm) was measured. The results are presented as a percentage of the control.

#### 2.9.2 Novel Object Recognition Test

To assess the cognitive function in the mice, we used the Novel Object Recognition Test (NORT) using Smart v2.5 (Panlab S.L.U., Barcelona, Spain) (Leger et al., 2013). The mice were trained for 2 days and tested for 1 day; thus, total protocol time was 3 days. Briefly, on the first day, the mice explored the maze for 10 min in the apparatus (35 × 35 × 35 cm^3^) to adapt maze. The second day (i.e., after 24 h), two of the same objects, plastic rectangular column objects (familiar), were placed at 15 cm intervals in this maze and the mice were allowed to explore the objects for 5 min. The third day, one object was replaced with a glass circular column object (novel), and both the familiar and novel object were placed in the maze. The mice were allowed to explore the maze again for 5 min. The time spent for exploring the familiar and novel objects was measured; the results are presented as the discrimination index.

Discrimination index=(novel object time–familiar object time)/total time

#### 2.9.3 Passive avoidance test

To assess the short-term memory of the mice, we used the passive avoidance test (PAT) using MED-PC (Med associates Inc., St. Albans, England) (Eagle et al., 2016). The mice were trained and tested for 1 day each; thus, total protocol time was 2 days. Briefly, on the first day of the test, the mice were introduced to a chamber that was divided into light and dark zones by a gate. The mice were first allowed to explore the light zone for 60 s; they were then moved to the dark zone *via* the gate, where they received an electric shock (0.5 mA, 3 s). The next day, the latency of the mice in the dark zone was measured (maximum time, 180 s). The results are presented as a percentage of the control.

### 2.10 Brain tissue preparation

After the PAT test, mice anesthetized with isoflurane received an intracardial perfusion with PBS; and their brains were removed. For biochemical analyses, the hippocampus tissue was separated and restored at −80°C. For immunohistochemical analyses, the brain was subjected to post-fixation with 4% paraformaldehyde in PBS and immersed in an optimal cutting temperature compound at −80°C for cryoprotection. Then the hippocampal region was coronally sliced to 30 µm-thick sections.

### 2.11 ACh and acetylcholinesterase activity assay

To measure the neurotransmitter levels, we evaluated the ACh and acetylcholinesterase (AChE) expression in the mice hippocampi using the Amplex Red Acetylcholine/Acetylcholinesterase Assay Kit (cat. A12217). Briefly, the mouse hippocampi were lysed and the same amount of protein was allowed to react with a working solution (comprising the Amplex Red reagent, HRP, choline oxidase, and AChE) for 90 min in the dark. The resulting fluorescence was measured using SpectraMax i3 (Molecular Devices). The results are expressed as a percentage of the control.

### 2.12 Western blotting

The cells and the mouse hippocampus tissue were homogenized with a RIPA lysis buffer and incubated for 20 min. After incubation, the homogenate was centrifuged (12,000 rpm) at 4°C for 20 min, and the resulting supernatant was collected. Equal amounts of protein samples and separated on a 10% polyacrylamide gel *via* sodium dodecyl sulfate-polyacrylamide gel electrophoresis; the separated proteins were transferred to a PVDF membrane, which was blocked with a 3% BSA solution. Thereafter, the membrane was incubated in a 5% BSA solution overnight at 4°C with the following primary antibodies: anti-β-actin (sc-47778), Bcl-2 (#3498), HO-1 (#82206), NeuN (MAB377), Nrf2 (NBP1-32822), mBDNF (NB100-98682), phosphorylated-CREB (pCREB; #9198), phosphorylated pERK (pERK; #9101), ERK (#9102), and choline acetyltransferase (ChAT; ab181023). The membranes were washed and incubated with HRP-conjugated secondary antibodies (A90-137P or A120-108P) for 1 h at room temperature and then treated with an ECL solution (Pierce, Rockford, IL, United States). Immunoreactivity was recorded using a digital imaging system (Q9 Alliance; UVITEC Ltd., England, United Kingdom). The results were quantified using the ImageJ 1.52v software and normalized to β-actin.

### 2.13 Nissl staining

Nissl staining was performed to measured damaged neuronal cells; it is a classic nucleic acid staining method for nervous tissue. The frozen slices were stained with a 0.1% cresyl violet solution for 5 min and then washed with distilled water. They were then dehydrated with 70%, 90%, 95%, and 100% alcohol and cleared with xylene twice for 10 min. The slices were mounted with mounting medium and analyzed using a microscope (BX53F; Olympus Corporation, Tokyo, Japan). Another researcher counted blindly the number of damaged cells in hippocampus throughout total four regions (each two regions in the cornu ammonis (CA) 1 and 3.

### 2.14 Immunofluorescence staining

The slices were incubated with a blocking solution for 1 h at room temperature; they were then incubated with primary antibodies to anti-pERK, BDNF, and NeuN at 4°C overnight. After washing with PBS with tween-20 (PBST), the slices were incubated with a fluorescent secondary antibody (Invitrogen) at room temperature in the dark for 2 h and washed with PBST. All slices were mounted with mounting medium (Vector Laboratories, Inc.) and observed under 10× or ×20 magnification using a microscope (Lion Heart FX; Agilent, Santa Clara, CA, United States). We counted the number of pERK^+^/NeuN^+^ and BDNF^+^/NeuN^+^ cells in CA1 and CA3 of the hippocampus.

### 2.15 Data analysis

All data were analyzed using Prism 5.0 (GraphPad Software, San Diego, CA) and are expressed as mean ± standard error of the mean (SEM). Statistical comparisons of more than two groups were performed using a one-way analysis of variance, followed by the Student–Newman–Keuls *post-hoc* test. *p* < 0.05 was considered statistically significant.

## 3 Results

### 3.1 HPLC-CAD analysis of YG

**FIGURE 2:**
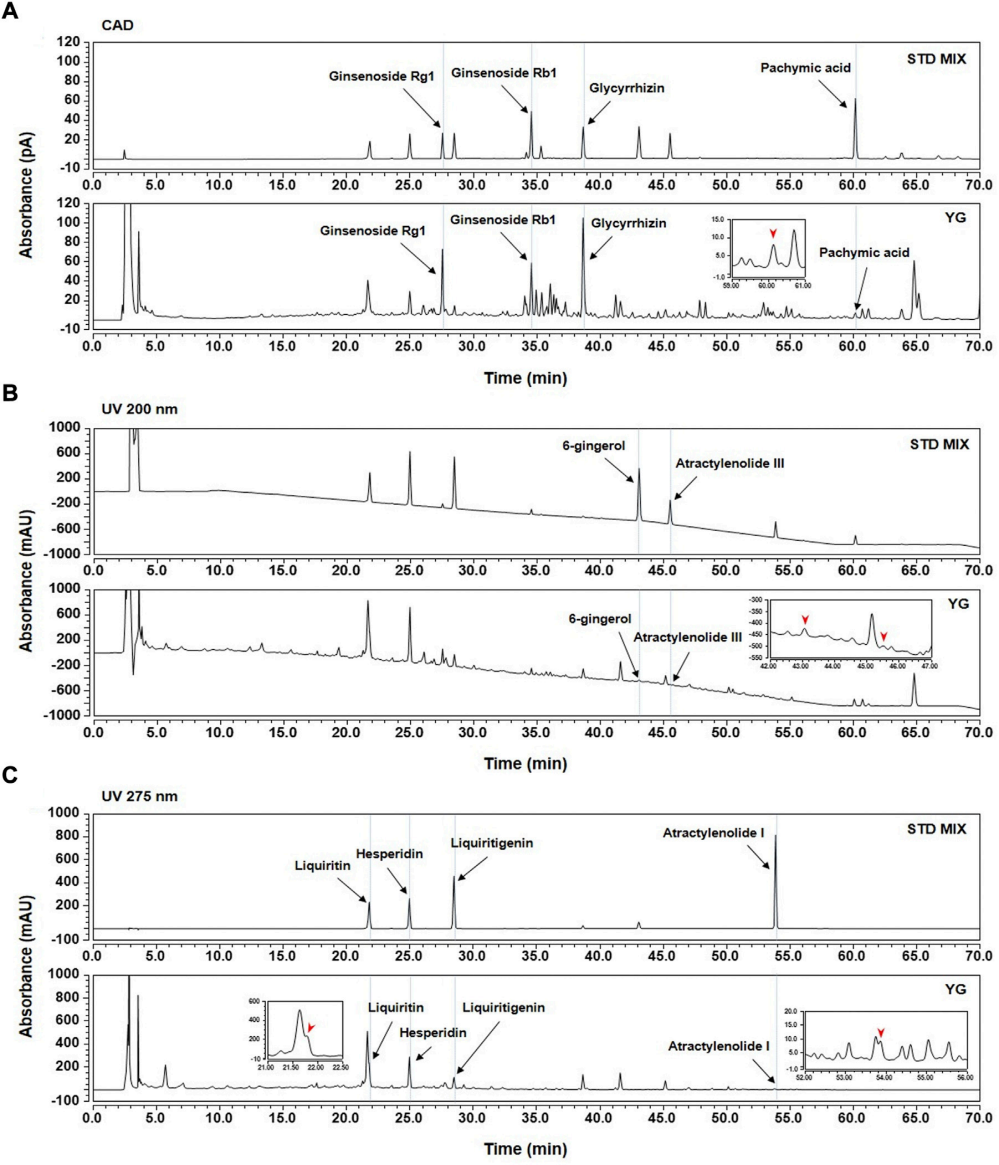
HPLC-CAD analysis of ten standards in YG. **(A)** CAD; **(B)** 200 nm; **(C)** 275 nm.

To identify the main components of YG, we selected the following ten compounds as reference standards for an HPLC-CAD analysis: ginsenoside Rb1 and Rg1 (*P. ginseng*); atractylenolide I and III (*A. macrocephala*); pachymic acid (*P. cocos*); hesperidin (*C. aurantium*); glycyrrhizin, liquiritin, and liquiritigenin (*G. uralensis*); and 6-gingerol (*Z. officinale*). Each compound was identified by comparing its retention time (*t*_R_), UV spectra, and chromatogram pattern with those of the reference standards using the HPLC system (Supplementary Figure S1). As shown in Figure 2, the *t*_R_s of the standard compounds were analyzed at 21.80 min (liquiritin; 275 nm), 24.97 min (hesperidin; 275 nm), 27.56 min (ginsenoside Rg1; CAD), 28.47 min (liquiritigenin; 275 nm), 34.58 min (ginsenoside Rb1; CAD), 38.65 min (glycyrrhizin; CAD), 43.06 min (6-gingerol; 200 nm), 45.52 min (atractylenolide III; 200 nm), 53.86 min (atractylenolide I; 275 nm), and 60.15 min (pachymic acid; CAD), which were compared with YG mixture under the same conditions. The *t*_R_s of the observed analytes in YG were 21.78 min (liquiritin; 275 nm), 24.96 min (hesperidin; 275 nm), 27.55 min (ginsenoside Rg1; CAD), 28.47 min (liquiritigenin; 275 nm), 34.58 min (ginsenoside Rb1; CAD), 38.65 min (glycyrrhizin; CAD),43.06 min (6-gingerol; 200 nm), 45.50 min (atractylenolide III; 200 nm), 53.85 min (atractylenolide I; 275 nm), and 60.14 min (pachymic acid; CAD).

### 3.2 Protective effect of YG against glutamate-induced cytotoxicity and apoptosis in HT-22 cells

**FIGURE 3:**
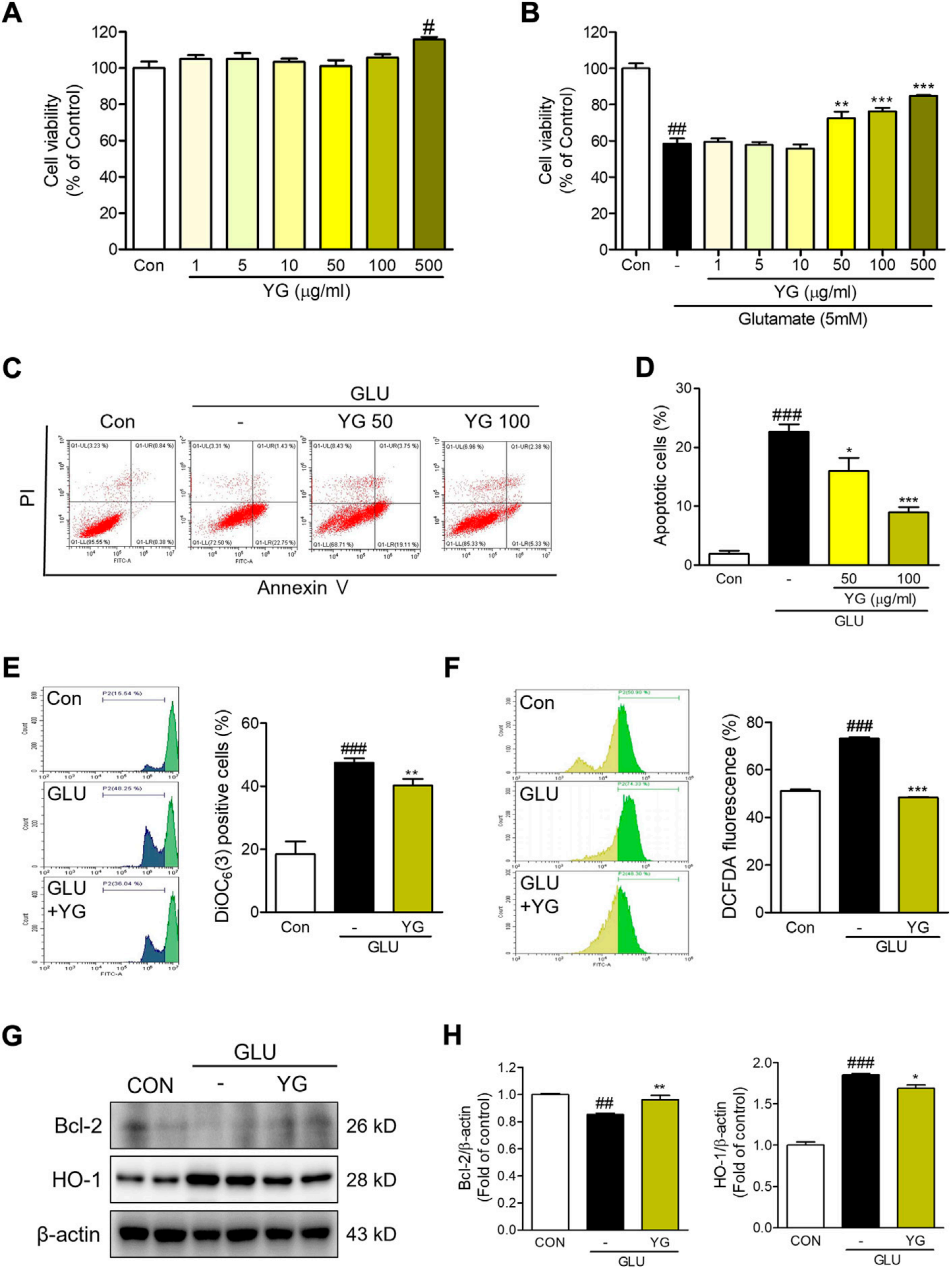
Neuroprotective effects of YG against glutamate-induced cytotoxicity in HT22 cells. **(A**,**B)** Cell viability. The results are calculated as a percentage of result values in control. **(C)** Representative flow cytometry analysis and **(D)** histogram for annexin V/PI staining. Representative flow cytometry analysis and histogram for **(E)** DiOC6(3) and **(F)** carboxy-H_2_DCFDA. **(G)** Western blot and **(H)** its densitometric analysis for Bcl-2 and HO1. All data are presented as the mean ± SEM from more than three independent experiments. ^#^ *p* < 0.05, ^##^ *p* < 0.01, ^###^ *p* < 0.001 vs. CON; ^*^ *p* < 0.05, ^**^ *p* < 0.01, ^***^ *p* < 0.001 vs. GLU. CON; control, GLU; glutamate 5mM, YG; Yuk-Gunja-tang 100 μg/ml.

To determine whether YG protects neuronal cells against glutamate-induced cytotoxicity, we performed an MTT assay. We found that YG had no cytotoxic effects within a concentration range of 1–500 μg/ml (Figure 3A). In assessments of glutamate-induced cytotoxicity, 50 and 100 μg/ml of YG treatment increased the cell viability (Figure 3B). To determine whether YG reduced glutamate-induced apoptosis in the HT22 cells, we performed an annexin V/PI staining. Glutamate treatment increased the population of apoptotic cells, while YG treatment (50 and 100 μg/ml) significantly reduced apoptosis in comparison with that in glutamate-treated cells (Figure 3C). Because the most effective concentration was found to be 100 μg/ml, we performed the subsequent experiments using this dose. These results show that YG treatment increased cell survival and reduced apoptosis in the hippocampal neuronal cells.

### 3.3 Effects of YG on oxidative stress and mitochondrial dysfunction in glutamate-induced HT-22 cells

To determine the effects of YG on mitochondrial dysfunction, we performed a DiOC_6_(3) staining. We found that the DiOC_6_(3) levels in glutamate-treated cells were markedly higher than those in the control cells. The DiOC_6_(3) levels in YG-treated cells were significantly lower than those in the glutamate-treated cells (Figure 3D). To determine the effects of YG on glutamate-induced oxidative stress, reactive oxygen species (ROS) generation was assessed using carboxy-H_2_DCFDA. We found that DCF fluorescence was increased in the glutamate-treated cells; YG treatment reduced the DCF fluorescence levels in comparison with the levels observed after glutamate treatment (Figure 3E). To confirm the neuroprotective effects of YG, we investigated the levels of proteins related to cell survival and antioxidant activity (Figure 3F). The expression of Bcl-2, an anti-apoptotic protein in mitochondria, was reduced by glutamate treatment and increased by YG treatment. In addition, the expression of HO-1, an antioxidant protein, increased with glutamate treatment and reduced with YG treatment (Figure 3G). These results show that YG treatment decreased ROS accumulation and mitochondrial dysfunction in the hippocampal neuronal cells.

### 3.4 Neuroprotective effects of YG *via* regulation of ERK, CREB, and BDNF

**FIGURE 4:**
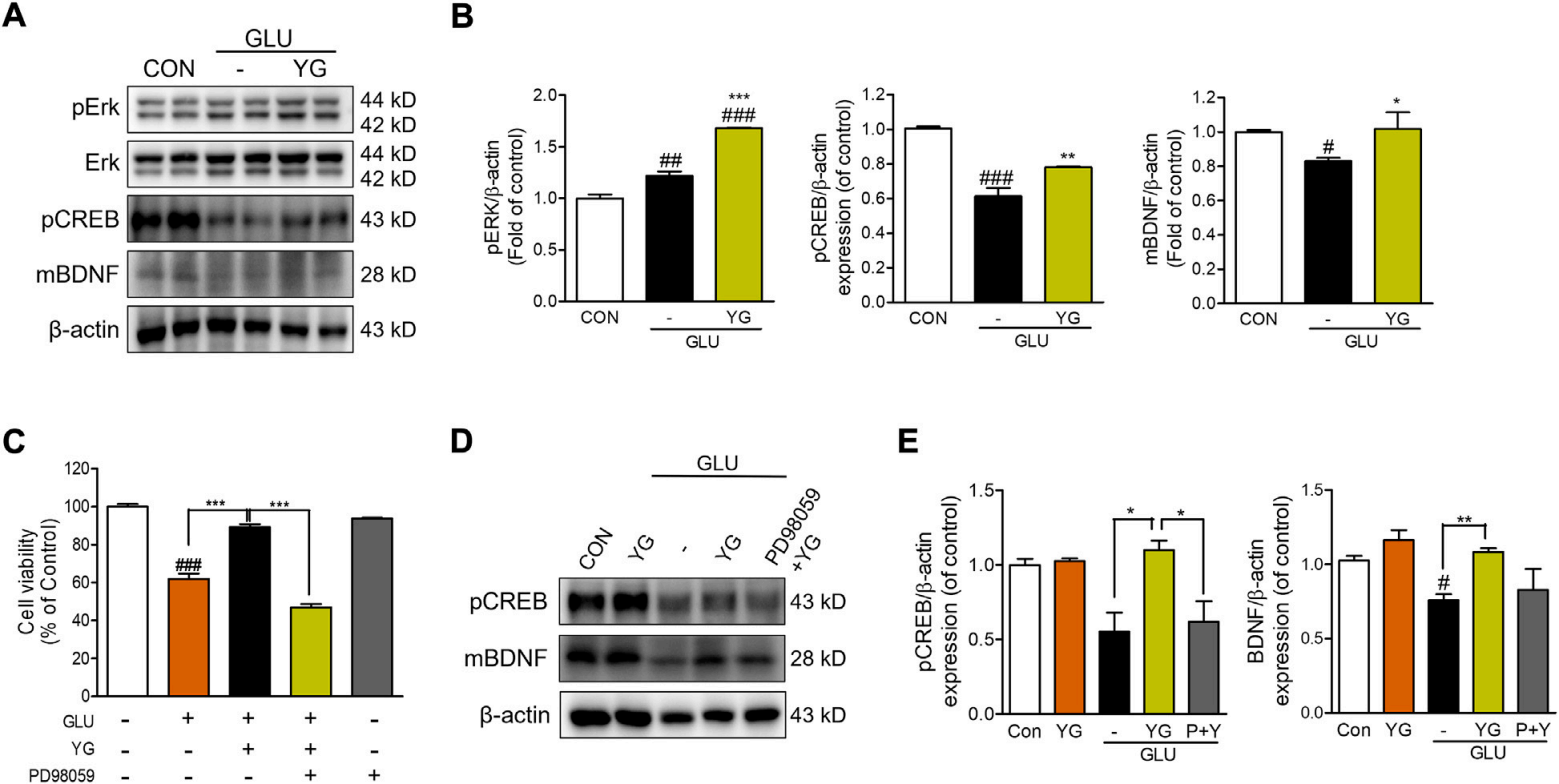
YG regulates ERK, CREB and BDNF activation in glutamate-induced cytotoxicity in HT22 cells. **(A)** Western blot band and **(B)** its histogram for pERK, pCREB and mBDNF. **(C)** Cell viability of ERK inhibitor, PD98059 with YG treatment. **(D)** Western blot band and **(E)** its histogram for pCREB and mBDNF in glutamate-induced cells treated YG and/or PD98059 (10 μM). All data are presented as the mean ± SEM from three independent experiments. ^#^ *p* < 0.05, ^##^ *p* < 0.01, ^###^ *p* < 0.001 vs. CON; ^*^ *p* < 0.05, ^**^ *p* < 0.01, ^***^ *p* < 0.001 vs. GLU. CON; control, GLU; glutamate 5mM, YG; Yuk-Gunja-tang 100 μg/ml, P + Y; PD98059 + YG.

Previous studies have shown that ERK, CREB, and BDNF activation promotes neuroprotective effects (Wang et al., 2018). Therefore, we investigated whether YG activated these signaling pathways (Figure 4). The expression of pERK in glutamate-treated cells was significantly greater than that in the control cells. In YG-treated cells, the expression of pERK was markedly greater than that in the control and glutamate-treated cells. We also found that glutamate treatment decreased the expression of pCREB and BDNF, which was increased by YG treatment (Figures 4A,B). To determine whether the neuroprotective effect of YG is mediated by activation of the ERK signaling pathway, we investigated the effects of YG on cell viability in the presence of an ERK chemical inhibitor (PD98059). PD98509 treatment (10 μM) dramatically abrogated restoration of cell viability by YG treatment against glutamate-induced cytotoxicity (Figure 4C). In addition, we also investigated whether activation of CREB/BDNF signaling by YG treatment is mediated *via* ERK signaling using PD98059 (Figure 4D). As expected, YG treatment increased the expression of pCREB and BDNF under glutamate-induced cytotoxic conditions. However, PD98059 treatment reversed the YG-induced increases in pCREB and BDNF expression (Figure 4E). These results showed that the neuroprotective effect of YG is mediated *via* the ERK, CREB, and BDNF signaling pathways in the hippocampal neuronal cells.

### 3.5 YG improves memory function in scopolamine-treated mice

**FIGURE 5:**
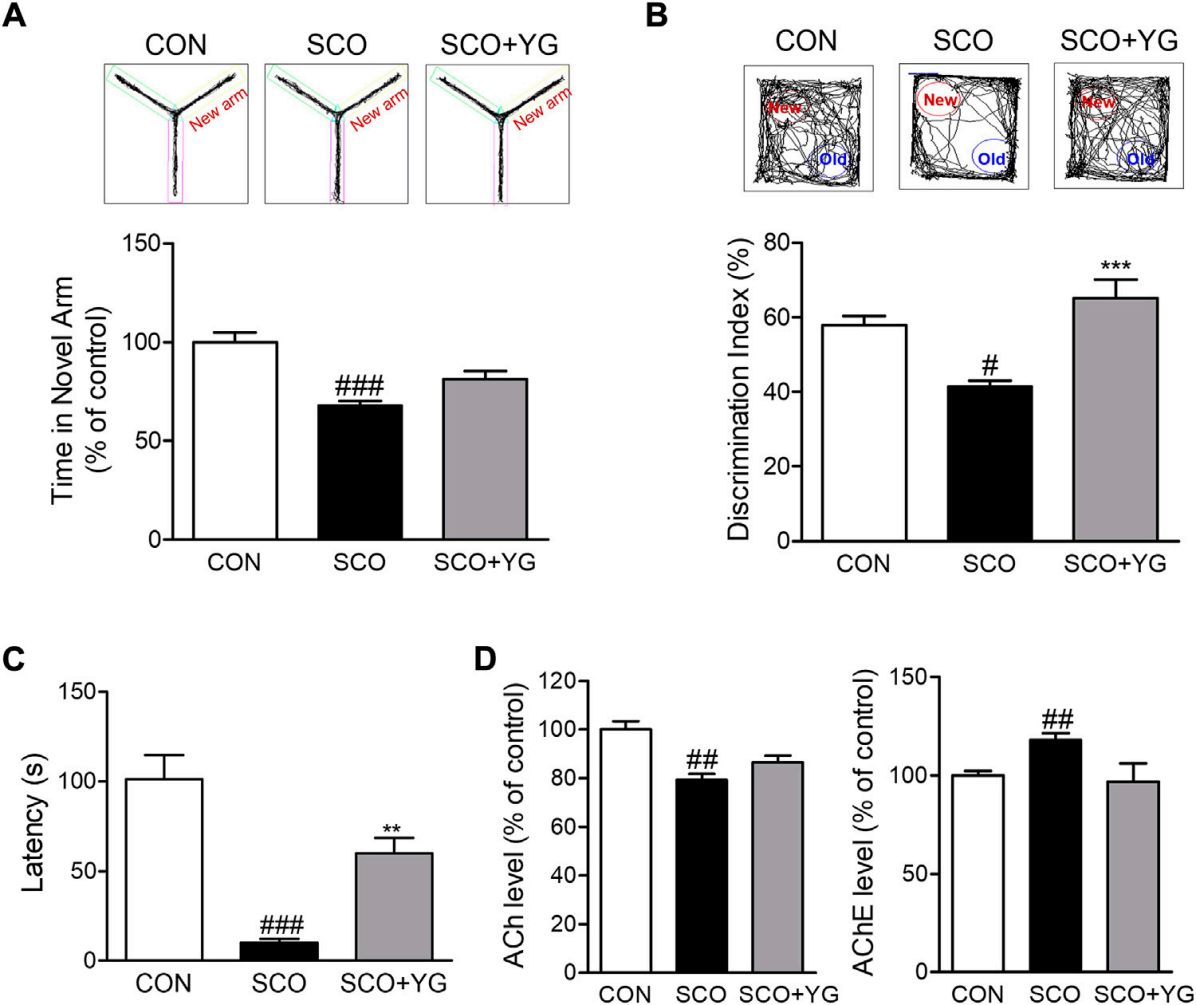
Effect of YG on memory impairment and acetylcholinergic signaling in scopolamine-induced mice. Behavior test in **(A)** Y-maze, **(B)** NORT and **(C)** PAT and **(D)** ACh and AChE level in the hippocampus tissue of mice. All data are presented as the mean ± SEM from more than three independent mice. ^#^ *p* < 0.05, ^##^ *p* < 0.01, ^###^ *p* < 0.001 vs. Con; ^*^ *p* < 0.05, ^***^ *p* < 0.001 vs. SCO. NORT; novel object recognitive test, PAT; passive avoidance test, ACh; Acetylcholine, AChE; Acetylcholinesterase, CON; control, SCO; scopolamine, SCO + YG; scopolamine + Yuk-Gunja-tang 150 mg/kg.

To determine whether YG treatment improved memory function, we performed the Y-maze test, NORT, and PAT to assess behavior-related cognition in a scopolamine-induced mouse model (Figure 5). In the Y-maze test, the mice in the SCO group spent less time in the novel arm than in the CON group; this time in the SCO + YG group was increased significantly (Figure 5A). In the NORT, the discrimination index to identify a novel apparatus in the SCO group was significantly lower than in the CON group; mice in the SCO + YG group showed a recovery index similar to that shown by the CON group (Figure 5B). In the PAT, the time to the dark zone in the SCO group was markedly shorter than in the CON group; mice in the SCO + YG group increased this time in the SCO group (Figure 5C). Moreover, we found the decreased ACh and AChE levels in SCO group; these levels in the SCO + YG group tended to recovery (Figure 5D). These results show that YG treatment improved memory function and acetylcholinergic system recovery in a scopolamine-induced mouse model.

### 3.6 YG increases neuronal cell survival in the hippocampi of scopolamine-induced mice

**FIGURE 6:**
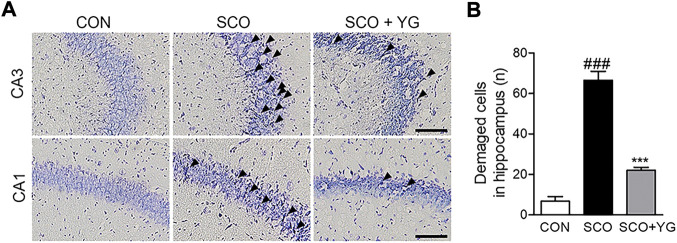
Effect of YG on neuronal cell death in scopolamine-induced mice. **(A)** Photomicrograph and **(B)** histogram for Nissl staining. All data are presented as the mean ± SEM from three independent mice. Scale bar = 100 μm ^###^ *p* < 0.001 vs. CON; ^***^ *p* < 0.001 vs. SCO. CON; control, SCO; scopolamine, SCO + YG; scopolamine + Yuk-Gunja-tang 150 mg/kg, CA1; cornu ammonis1, CA3; cornu ammonis3.

We performed Nissl staining of tissues from the hippocampi of scopolamine-treated mice to determine whether YG inhibited neuronal cell death (Figure 6). The number of damaged neuronal cells in the hippocampus of SCO group increased and these cells decreased significantly in the SCO + YG group (Figure 6B). These results showed that YG treatment decreased neuronal cell death in the hippocampi of scopolamine-treated mice.

### 3.7 YG recovers neuronal function and activates ERK signaling in the hippocampi of scopolamine-induced mice

**FIGURE 7:**
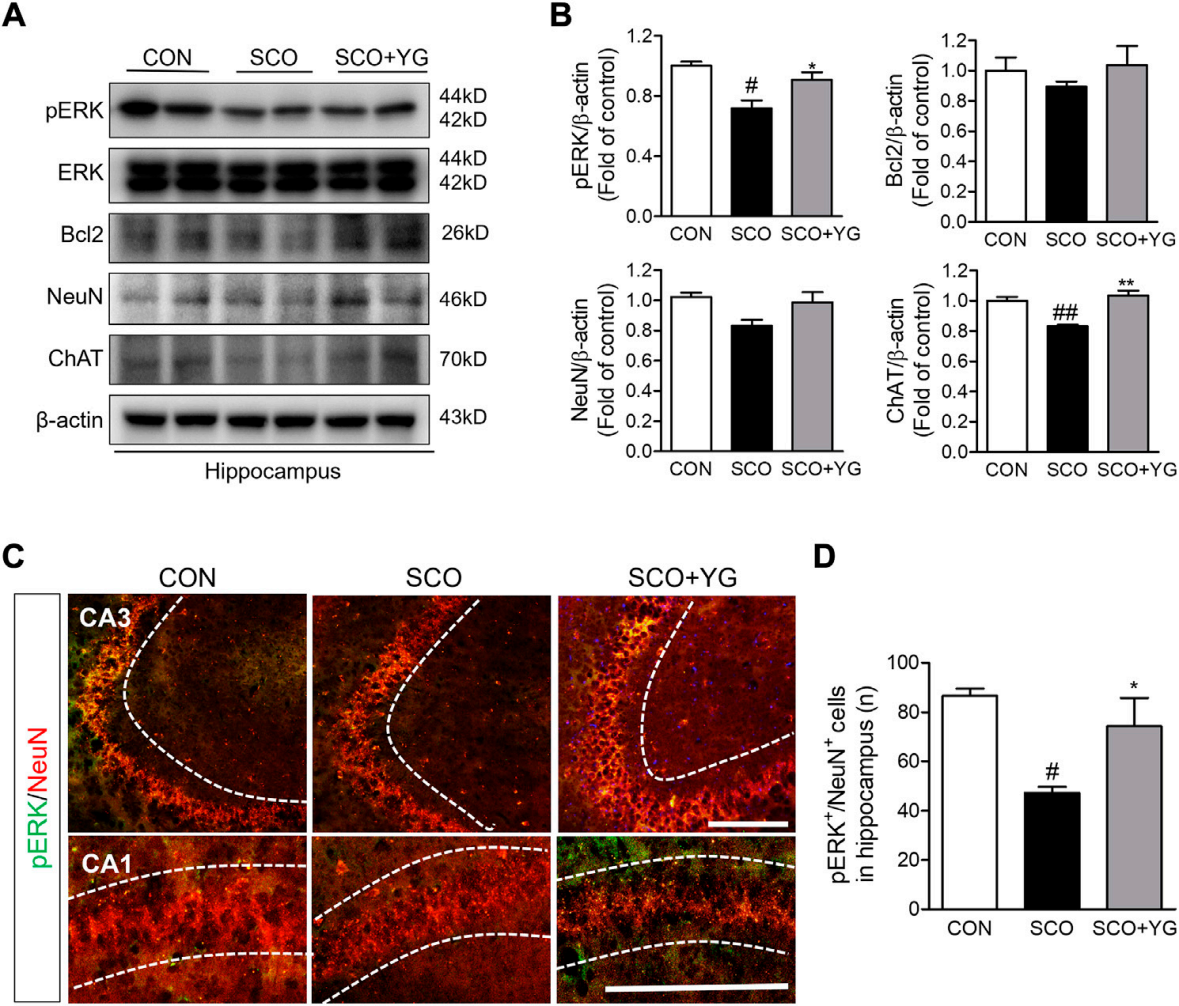
Effects of YG on ERK signaling-related protein expression in hippocampus of mice. **(A)** Western blot and **(B)** its histogram for Bcl2, ChAT, NeuN and pERK. Histograms show protein relative to β-actin protein. Double-immunostaining of pERK and NeuN in hippocampus of mice. **(C)** Microphotographs and **(D)** histogram for pERK and NeuN in the CA1 and 3 regions. All data are presented as the mean ± SEM from three independent mice. ^#^ *p* < 0.05, ^##^ *p* < 0.01, vs. CON; ^*^ *p* < 0.05, ^**^ *p* < 0.01 vs. SCO by ANOVA with Newman-keuls post-hoc analysis. Scale bar = 200 μm. CON; control, SCO; scopolamine, SCO + YG; scopolamine + Yuk-Gunja-tang 150 mg/kg, CA1; cornu ammonis1, CA3; cornu ammonis3.

*In vitro* results showed that YG treatment decreased neuronal death and activated ERK signaling. Therefore, we investigated the effects of YG on neuronal survival and ERK signaling in the hippocampi of scopolamine-treated mice (Figure 7). Bcl-2, ChAT and NeuN expressions in the hippocampal tissue were lower in the SCO group than in the CON group; these were significantly increased following YG treatment (not significant in Bcl-2 and NeuN expression). Furthermore, the level of pERK expression was significantly higher in the SCO + YG group than in the CON and SCO groups (Figure 7B). We also examined pERK expression in the mouse hippocampus region by immunofluorescence staining (Figures 7C,D); the number of pERK^+^/NeuN^+^ cells in the SCO group was lower than that in the CON group, while the number of pERK^+^/NeuN^+^ cells in the SCO + YG group was higher than that in the SCO group (Figure 7D). These results showed that YG treatment decreased neuronal cell death in the hippocampi of scopolamine-treated mice.

### 3.8 YG activates CREB and BDNF signaling in the hippocampi of scopolamine-induced mice

**FIGURE 8:**
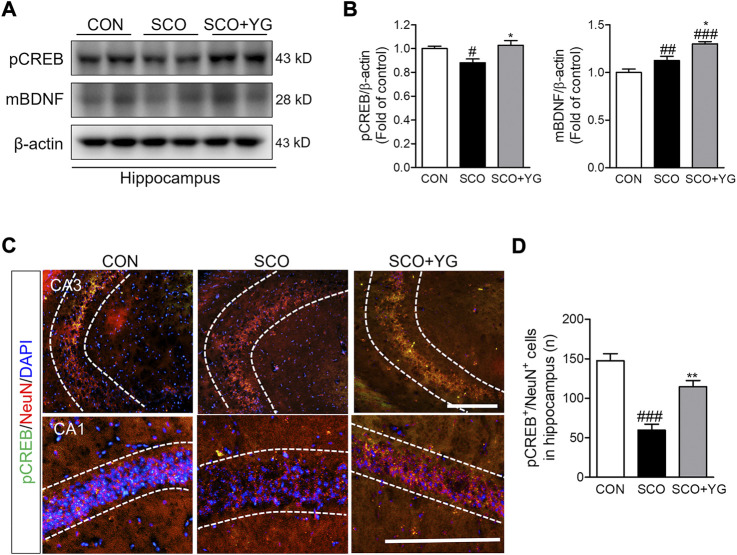
YG regulate CREB/BDNF signaling in hippocampus of mice. **(A)** Western blot and **(B)** its histogram for pCREB and mBDNF. Histograms show protein relative to β-actin protein. Double-immunostaining of pCREB and NeuN in hippocampus of mice. **(C)** Microphotographs and **(D)** histogram for pCREB and NeuN in the CA1 and 3 regions. All data are presented as the mean ± SEM from three independent mice. ^#^ *p* < 0.05, ^##^ *p* < 0.01, ^###^ *p* < 0.001 vs. CON; ^*^ *p* < 0.05, ^**^ *p* < 0.01 vs. SCO by ANOVA with Newman-keuls post-hoc analysis. Scale bar = 200 μm. CON; control, SCO; scopolamine, SCO + YG; scopolamine + Yuk-Gunja-tang 150 mg/kg; CA1; cornu ammonis 1, CA3; cornu ammonis 3.

We investigated whether YG treatment activated CREB/BDNF signaling in the hippocampi of scopolamine-treated mice. The level of pCREB expression was lower in the SCO group than in the CON group; it increased significantly following YG treatment. The level of mBDNF expression was significantly higher in the SCO + YG group than in the CON and SCO groups (Figure 8B). The number of pCREB^+^/NeuN^+^ cells in the hippocampal tissue was higher in the SCO group than in the CON group; YG treatment increased this number in the CA region significantly (Figures 8C,D). These results showed that YG treatment regulated CREB/BDNF and ERK/CREB/BDNF signaling in the hippocampi of scopolamine-treated mice.

## 4 Discussion

**FIGURE 9:**
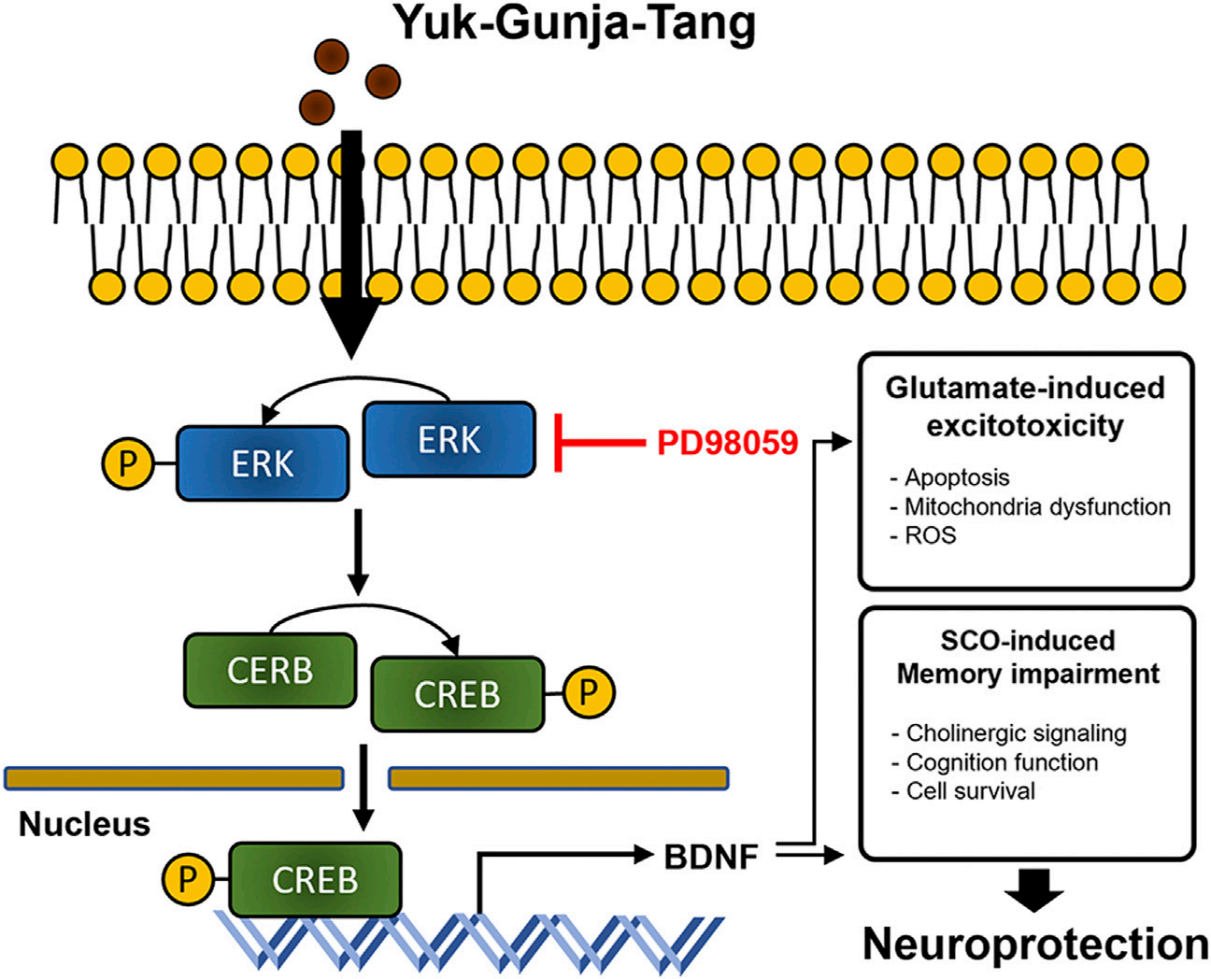
Diagram showing a hypothesized mechanism by Yuk-Gunja-Tang is effective in the treatment of Alzheimer’s disease. ERK: extracellular signal-regulated kinase, CREB: cAMP response element binding protein, BDNF: brain derived neurotrophic factor, ROS: reactive oxygen species, SCO: scopolamine.

YG treatment significantly restored cell viability to suppress glutamate-induced ROS accumulation and mitochondrial dysfunction in the HT22 cells. YG increased activation of ERK, CREB and BDNF under glutamate-induced excitotoxic conditions. Additionally, in the scopolamine-induced cognitive impairment mouse model, YG restored cognition and acetylcholine and reduced neuronal damage. YG also increased activation of ERK, CREB and BDNF in the hippocampus of scopolamine-induced mice (Figure 9).

AD is characterized by progressive cognitive decline and neuronal loss, which are associated with oxidative stress in the brain (Cobley et al., 2018). Currently, donepezil and memantine are used to delay the progression of AD symptoms. It is limitation in clinical outcomes of AD drugs, and they also have dangerous side effects such as vasodilation, headache, and convulsions (Corbett et al., 2013). Therefore, therapy of AD with natural products is potentially useful and safe (Habtemariam, 2019). YG, which consists of eight botanical drugs, has been used as a traditional medicine for treating gastrointestinal dysfunction (Saegusa et al., 2015). Previous studies have reported that YG administration is more effective for functional dyspepsia (Xiao et al., 2012) and that YG has a regulatory effect on ghrelin signaling and receptors (Yamada et al., 2021). However, there are no studies on the neuroprotective effects of YG against neurodegenerative diseases as yet; therefore, ours is the first study to explore the neuroprotective effect of YG in the hippocampus of experimental AD model.

The hippocampus is important for both spatial and recognition memory and regulates many neurotransmitters involved in AD-associated cognitive dysfunction (including those mediating the glutamatergic and cholinergic networks) (Broadbent et al., 2004). Here, we used two experimental AD models for identifying the possible neuroprotective effect of YG in the hippocampus.

In the HT22 cells, an immortalized mouse hippocampal neuronal cell line, excessive glutamate stimulation evokes oxidative stress, mitochondrial dysfunction, and cell death through necrotic and apoptotic processes (Kritis et al., 2015), that is be components of AD (Hynd et al., 2004). We found that YG treatment enabled the most effective recovery of viability than treatment with single YG components (Supplementary Figure S2). In addition, YG decreased excitotoxicity by reducing apoptotic cell death, ROS generation, and mitochondrial dysfunction (Figures 2, 3). In our data, we observed that HO-1 expression is increased in glutamate-induced excitotoxicity. Depletion of cellular GSH triggers HO-1 expression (Ryter and Choi, 2002) and oxidative stress increased expression of HO-1, following increased apoptosis factors (Pietrofesa et al., 2016). It suggests HO-1 is an oxidative stress marker in glutamate-induced HT 22 cell (Figure 3).

A previous study has reported glutamate injection into the brain is used as experimental epilepsy model (Rubio et al., 2010), so we studied using scopolamine-induced AD model to confirm that YG improve symptoms of cognitive decline. Scopolamine blocks the binding of ACh to muscarinic receptors, which are predominantly located in the hippocampus (Klinkenberg and Blokland 2010). This causes cholinergic dysfunction and cognitive impairment (Chen and Yeong 2020). ACh is synthesized by ChAT and broken down by AChE. ChAT is upregulated during spatial memory tasks, while a decreased ACh level is associated with cognitive decline (Maurer and Williams 2017). Using scopolamine-induced AD-like animals, we found that YG administration improved spatial and learning memory (despite slight hyperactivity caused by scopolamine injection; Supplementary Figure S4) and reduced neuronal cell death in the hippocampus (Figure 5). Spatial and learning memory is associated with hippocampal function (Leger et al., 2013; Eagle et al., 2016; Kraeuter et al., 2019), suggesting that YG affects hippocampal function. Interestingly, we found that YG did not increase the ACh levels dramatically, but led to an improved memory function under scopolamine-induced conditions. This suggests that YG leads to cognitive improvement by regulating not only cholinergic system restoration, but also endogenous signaling.

ERK/CREB signaling mediates neurotrophic factors and is neuroprotective (Grimes et al., 2015). ERK signaling controls synaptic plasticity and memory (Kelleher et al., 2004); in the hippocampus, this pathway promotes passive avoidance memory retrieval (Kim et al., 2012). Activation of this signaling pathway involves the phosphorylation of CREB (Alberini, 2009; Deepa et al., 2020), which stimulates the expression of cell survival-related genes (such as Bcl-2) and growth factors (such as BDNF) (Sakamoto and Frank 2009). BDNF plays an important role in neuronal survival and memory processing (Huang and Reichardt, 2001; Bekinschtein et al., 2014). Previous studies have shown that ERK/CREB signaling is involved in neuroprotection in various brain injuries and scopolamine-induced memory impairment (Park et al., 2004; Atkins et al., 2009; Li et al., 2016; Deepa et al., 2020). A recent study reported that activation of the ERK/CREB/BDNF signaling pathway protects against scopolamine-induced memory deficits by reducing oxidative stress (Lv et al., 2021). Furthermore, CREB and BDNF signaling are attenuated by amyloid beta toxicity (Amidfar et al., 2020). In present study, YG treatment activated the ERK/CREB/BDNF signaling pathway, and experiments with the chemical inhibitor PD98059 revealed that the neuroprotective effect of YG is exerted through an ERK-dependent mechanism in the HT22 cells (Figure 4). Also, gene expression of BDNF was increased by YG treatment (not CREB gene expression; Supplementary Figure S3), which is suggest that CREB is phosphorylated by activation of ERK, and then promote BDNF transcription (Benarroch, 2015). Moreover, YG administration in the mouse model also activated ERK and CREB phosphorylation, which eventually increased the expression of BDNF in the hippocampal tissue. These studies suggest that YG activates the ERK/CREB/BDNF signaling pathway in the hippocampus under pathological conditions, potentially improving memory function (Figures 4, 7, 8).

The hippocampus proper is divided into the dentate gyrus (DG) and CA regions (Nava-Mesa et al., 2013). The CA1 and CA3 regions are associated with memory processes and are susceptible to neurodegeneration (Khakpai et al., 2012; Rolls, 2013; Cherubini and Miles, 2015). Additionally, the CA3 hippocampal synapse may account for memory deficits during the early stages of AD (Nava-Mesa et al., 2013). In our study (Figure 6), it was observed that the ERK/CREB signaling pathway was significantly altered in the CA3 region but not in the DG (data not shown). Therefore, YG treatment may exert a neuroprotective effect in the CA region of the hippocampus in AD.

Because individual botanical drugs of YG have neuroprotective properties (Supplementary Table S1), this may be the reason for neuroprotection of YG. In addition, our data that YG regulates oxidative stress, apoptosis, and ERK-BDNF signals supports this. It is also possible to control the effect of YG indirectly through other signaling. Previous studies have shown that YG regulates ghrelin signaling (Yamada et al., 2021), and ghrelin signaling associated with neurocognitive effects on the hippocampus (Seminara et al., 2018), which may be a novel signal for neuroprotection in YG. Furthermore, YG may penetrate directly into the brain and regulate these signaling. To determine which mechanism is used, the as-yet unknown bioavailability of YG should be investigated further. Additionally, the effects of YG in popular animal models of AD, such as the 3xTg or 5xFAD mice, have not yet been investigated. Another limitation of our study did not consider the mouse conversion factor 12.3 in a calculation of YG dose (Nair and Jacob, 2016). Interestingly, YG was effective even at our setting dose, indicating that YG attenuates cognitive decline at lower dose than that used for the treatment of functional dyspepsia. This suggests that low dose YG may be a merit for long-term treatment for patients with dementia.

In conclusion, YG exerts a protective effect by reducing cell death, oxidative stress, and mitochondrial dysfunction *via* ERK/CREB signaling in hippocampal cells under excitotoxicity. Furthermore, it also restores cholinergic signaling through the ERK/CREB/BDNF signaling pathway in the hippocampus, which is related to cognitive function in AD. Therefore, YG may have a therapeutic potential against cognitive impairment in neurodegenerative diseases.

## Data Availability

The original contributions presented in the study are included in the article/Supplementary Material, further inquiries can be directed to the corresponding author.
