# Supplementary material for: Yuk-Gunja-Tang attenuates neuronal death and memory impairment via ERK/CREB/BDNF signaling in the hippocampi of experimental Alzheimer’s disease model
Source: Front Pharmacol. 2022 Oct 26;13:1014840. doi: 10.3389/fphar.2022.1014840 (PMC9643579; doi:10.3389/fphar.2022.1014840)
Supplement: Supplementary file 1 [file DataSheet1.docx]

Supplementary Material

1. **Supplementary Material and Methods**

# 1.1 Preparation of standards and sample solution

Stock Solution

- Stock solutions of atractylenolide I, atractylenolide III, 6-gingerol, ginsenoside Rb1, ginsenoside Rg1, glycyrrhizin, liquiritin, liquiritigenin and pachymic acid were prepared by dissolving in 100% methanol at a concentration of 1000 μg/mL.
- Hesperidin was dissolved in 50% DMSO methanol solution at a concentration of 1000 μg/mL.
- Stock solutions of reference standards were each prepared at a concentration of 1000 μg/mL in methanol. Exceptionally, hesperidin was prepared in 50% DMSO methanol solution.

Working Solution

- 100 μg /mL in methanol: all components
- Then, 10 stock solutions were diluted to a final concentration of 100 μg/mL of each component to prepare a mixed working solution.

Analytical Samples

- 50.0 g of Yuk-gunja-tang (YG) decoction powder was extracted with 200 mL of methanol by reflux extraction for 3 hours (hereinafter, YG methanol extract; yield 3.58%). Then, the YG methanol extract was prepared in methanol at a concentration of 100 mg/mL and used as an analytical sample.

**1.2 Cell viability of YG decoction or single YG component in glutamate-induced excitotoxicity**

Preparation of single compound of YG

- We used YG decoction powder as made by Hankook Sinyak Corp. (water extract, yield: 15 %). The composition of YG is 8 plants (herbs), and that was obtained from Omniherb Corp. (Daegu, South Korea). They were immersed in 1,000 ml of water and extracted for 2 h 30 min by heating. The extract was subsequently filtered and lyophilized for 1 week. The final amounts of extracted following as:

| Scientific name | Part | Weight  (dried plant material, g) | Yield (%) |
| --- | --- | --- | --- |
| *Panax ginseng* C.A. Mey. | Root | 50.0 | 20.92 |
| *Atractylodes macrocephal* Koidz. | Root and rhizome | 50.0 | 27.48 |
| *Poria cocos* Wolf | Sclerotium | 50.0 | 0.82 |
| *Pinellia ternata* (Thunb.) Makino | Tuber | 50.0 | 6.48 |
| *Citrus × aurantium* f. *deliciosa*  (Ten.) M.Hiroe | Fruit peel | 50.0 | 19.98 |
| *Glycyrrhiza uralensis* Fisch. Ex DC. | Root and rhizome | 50.0 | 13.24 |
| *Zingiber officinale* Roscoe | Rhizome | 50.0 | 16.54 |
| *Ziziphus jujuba* Mill | Fruit | 50.0 | 11.76 |

Cell viability assay

- HT22 cells were cultured at 3 × 10^3^ cells/well in 96-well plate and incubated for 24 h. After incubation with100 μg/ml concentration of YG or YG component for 24 h, the cells were further incubated with glutamate (5 mM) treatment for 24 h. For determination of cell viability, the MTT assay was used. After all treatment, 10% MTT solution (5 mg/mL) was changed into the plate. Following incubation in the dark for 2 h at 37℃, the absorbance was determined at 540 nm using a microplate reader (Spectra-Max i3, Molecular devices, Sunnyvale, CA, USA). Results are expressed as a percentage of the control cells.

**1.3 Real-time Quantitative reverse transcription polymerase chain reaction (RT-qPCR)**

- After treatment, total RNA in HT22 cells was isolated using the RNeasy Mini, RNA isolation kit (Qiagen, Chatsworth, CA) according to manufacturer's protocol. Extracted RNA was synthesized into cDNA using Omniscript Reverse Transcriptase (Qiagen). SYBR green-based quantitative PCR amplification was performed using the QuantStudio 6 Flex Real-time PCR System (Thermo Scientific). The relative quantitation values of respective mRNAs were normalized to that of the endogenous β-actin control and were quantified by 2–ΔΔCt method. The set of primers were used to amplify mouse specific products blow to:

| Gene | Primer | Sequence |
| --- | --- | --- |
| Bdnf | Forward  Reverse | 5’- CGA CAT CAC TGG CTG ACA CT -3’  5’- CAA GTC CGC GTC CTT ATG GT -3’ |
| Creb | Forward  Reverse | 5’-TACCCAGGGAGGAGCAATAC-3’  5’-GAGGCAGCTTGAACAACAAC-3’ |
| β-actin | Forward  Reverse | 5’-GGCACCACACCTTCTACAATGA-3’  5’-ATCTTTTCACGGTTGGCCTTAG-3’ |

**2. Supplementary Figures**

-
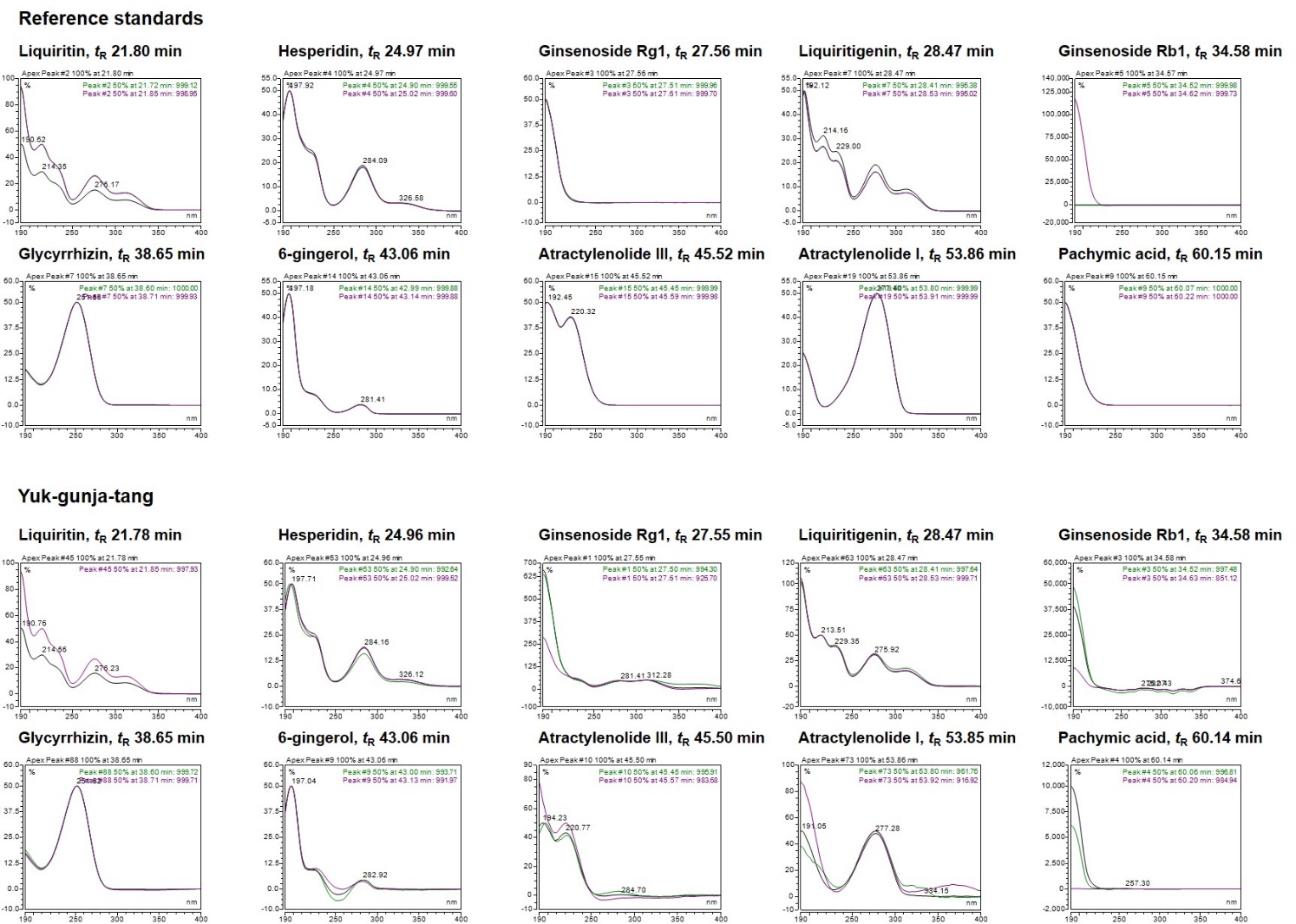


**Supplementary Figure 1**. UV spectra of 10 standards and YG extracts.

**Supplementary Figure 2.** Cell viability of YG decoction or single YG component on glutamate-induced excitotoxicity in HT22 cells. The single treatment of D or YG were recovery cell viability compared with glutamate treatment. Most effective recovery of viability was showed YG treatment than other single treatment. All data are expressed as the mean ± SEM. ^###^*P* < 0.001 vs. Con; ^*^*P* < 0.05, ^**^*P* < 0.01 vs. Glutamate. N = 6. Con: control; A: *Panax ginseng* C.A. Mey; B: *Pinellia ternata (Thunb.)* Makino; C: *Atractylodes macrocephal* Koidzumi; D: *Poria cocos* Wolf; E: *Citrus × aurantium* f. *deliciosa* (Ten.) M.Hiroe; F: *Glycyrrhiza uralensis* Fisch; G: *Zingiber officinale* Roscoe; H: *Ziziphus jujuba* Mill; YG: Yuk-gunja-tang.

**Supplementary Figure 3**. Gene expression of YG treatment in glutamate-treated HT22 cells. The gene expression of Bdnf was significantly increased by YG treatment compared with levels in the glutamate treatment. All data are expressed as the mean ± SEM. ^#^*P* < 0.05 vs. CON; ^**^*P* < 0.01 vs. GLU. N = 3. CON; control, GLU; glutamate.


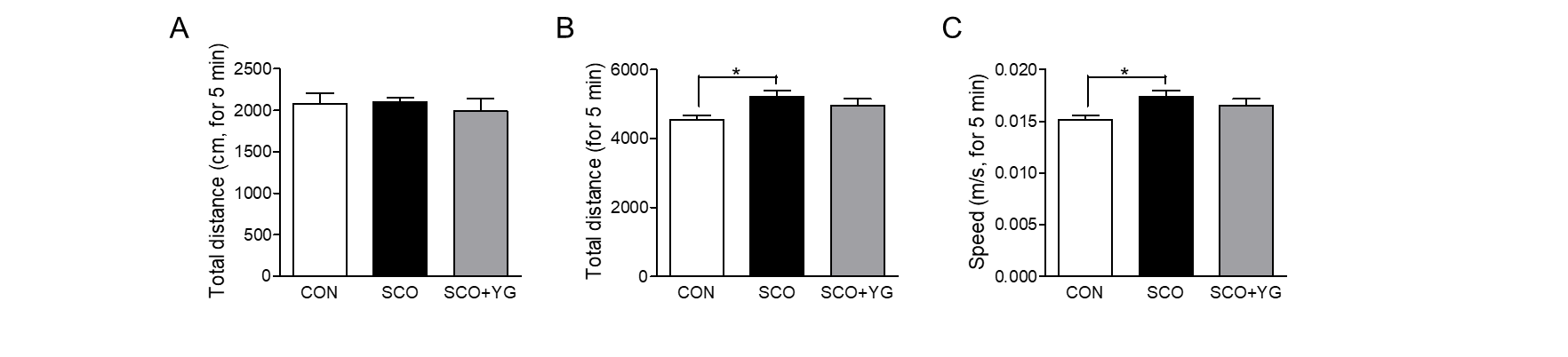


**Supplementary Figure 4**. Behavior tests in (A) Y-maze and (B, C) novel objects recognition test (NORT). Total distance travelled in the (A)Y-maze and (B) NORT maze. (C) Speed of mice in NORT test. Gene expression of YG treatment in glutamate-treated HT22 cells. All data are presented as the mean ± SEM from six independent mice. ^*^*P* < 0.05, CON; control, SCO; scopolamine, SCO+YG; scopolamine + Yuk-Gunja-tang 150 mg/kg.

1. **Supplementary Table 1.**

The neuroprotection in individual botanical drugs of Yuk-gunja-tang (YG).

| Botanical  drug name | Model | Signaling | PMID  or Doi |
| --- | --- | --- | --- |
| *Panax Ginseng* | HT22 cell (glutamate)  Mice (LPS-induce deficit)  Mice (scopolamine) | MAPK signaling  (Ginsenoside Rb2)  Erk-BDNF signaling  Anti-neuroinflammatory  (polysaccharides) | 30976171  33841001 |
| *Pinellia ternata* | Ellman’s method and modified TLC bioautographic assay  Rats (ischemia-reperfusion) | AChE inhibitory activity  Anti-inflammatory and apoptosis  (n-butyl alcohol extracts) | Doi:10.1007/s00044-011-9582-8  27132713 |
| *Atractylodes macrocephala* | Cortical neuron (hypoxia)  SH-SY5Y cell (MPP^+^) | Inhibition neuronal apoptosis  (polysaccharides)  None  (new compounds) | 24718967  28027699 |
| *Poria cocos* | HT22 cell (H_2_O_2_)  Rats (D-galactose)  B35 and C6 cells (-) | None  (70% ethanol extract)  MAPK/NFκB  (polysaccharide)  Cell migration and actin filament  (water solution) | 29391873  33913927  34512781 |
| *Citrus × aurantium* f. *deliciosa* | HT22 cell (H_2_O_2_)  Mice (amyloid β)  PC12 cell (glutamate) | None  (70% ethanol extract)  Anti-apoptosis, cognition  (extract)  Anti-oxidant  (70% ethanol extract) | 29391873  31432129 |
| *Glycyrrhiza uralensis* | HT22 cell (glutamate)  BV2 cell (LPS)  PC12 cell (H_2_O_2,_, 6-OHDA)  BV2 cell (LPS) | Protect mitochondrial damage  (Isoliauiritigenin Compound)  None,  (Glycoside Compound)  inhibited TNF-α, elevated BDNF  (flavonoids) | 22538371  Doi:10.1177/1934578X21992988  Doi:10.1016/j.jtcms.2014.11.004 |
| *Zingiber officinale* | HT22 cell (H_2_O_2_)  Rats (acrylonitrile)  Rats (ischemia-reperfusion) | None  (70% ethanol extract)  Anti-inflammation  (6-Gingerol-rich fraction)  Anti-oxidant effect  (95% ethanol extract) | 29391873  30864424  21197427 |
| *Ziziphus jujuba* | Rats (scopolamine)  PC12 cell (H_2_O_2_)  Mice (amyloid β) | Anti-inflammatory and apoptosis  (Aqueous extract)  Anti-oxidant  (60% ethanol extract)  Anti-oxidant, cognition  (70% ethanol extract) | 34422074  Doi:10.1007/s10068-015-0296-4  33841722 |

**Reference**

Choi, J., An, X., Lee, B.H., Lee, J. S., Heo, H. J., Kim, T., Ahn, J., and Kim, D., 2015. Protective effects of bioactive phenolics from jujube (Ziziphus jujuba) seeds against H_2_O_2_–induced oxidative stress in neuronal PC-12 cells. *Food Sci. Biotechnol.* 24: 2219–2227. doi:10.1007/s10068-015-0296-4.

Djeuzong, E., Kandeda, A. K., Djiogue, S., Stéphanie, L., Nguedia, D., Ngueguim, F., Djientcheu, J. P., Kouamouo, J., and Dimo, T., 2021. Antiamnesic and Neuroprotective Effects of an Aqueous Extract of Ziziphus jujuba Mill. (Rhamnaceae) on Scopolamine-Induced Cognitive Impairments in Rats. *Evid. Based Complement. Alternat. Medi.: eCAM*, 5577163. doi:.10.1155/2021/5577163.

Farombi, E. O., Abolaji, A. O., Adetuyi, B. O., Awosanya, O., and Fabusoro, M., 2018. Neuroprotective role of 6-Gingerol-rich fraction of Zingiber officinale (Ginger) against acrylonitrile-induced neurotoxicity in male Wistar rats. *J. Basic Clin. Physiol. Pharmacol.* 30(3): 10.1515/jbcpp-2018-0114. doi:10.1515/jbcpp-2018-0114.

Kim, D. H., Kim, D. W., Jung, B. H., Lee, J. H., Lee, H., Hwang, G. S., Kang, K. S., and Lee, J. W., 2019. Ginsenoside Rb2 suppresses the glutamate-mediated oxidative stress and neuronal cell death in HT22 cells. J. Ginseng Res. 43(2): 326–334. [doi:10.1016/j.jgr.2018.12.002](https://doi.org/10.1016/j.jgr.2018.12.002).

Kim, M. J., Jung, J. E., Lee, S., Cho, E. J., and Kim, H. Y., 2021. Effects of the fermented Zizyphus jujuba in the amyloid β25-35-induced Alzheimer's disease mouse model. Nutr. Res. Pract. 15(2): 173–186. doi:10.4162/nrp.2021.15.2.173.

Kim, Y. J., Lim, H. S., Kim, B. Y., Seo, C. S., and Jeong, S. J., 2017. Quantitative Analysis and Biological Efficacies regarding the Neuroprotective and Antineuroinflammatory Actions of the Herbal Formula Jodeungsan in HT22 Hippocampal Cells and BV-2 Microglia. *Evid. Based Complement. Alternat. Medi.: eCAM*, 6360836. [doi:10.1155/2017/6360836](https://doi.org/10.1155/2017/6360836).

Lee, C. Y., Lee, C. T., Tzeng, I. S., Kuo, C. Y., Tsai, F. M., & Chen, M. L., 2021. Poria cocos Regulates Cell Migration and Actin Filament Aggregation in B35 and C6 Cells by Modulating the RhoA, CDC42, and Rho Signaling Pathways. *Evid. Based Complement. Alternat. Medi.: eCAM*, 6854860. doi:10.1155/2021/6854860.

Lee, H. J., Lee, S. K., Lee, D. R., Choi, B. K., Le, B., and Yang, S. H., 2019. Ameliorating effect of Citrus aurantium extracts and nobiletin on β‑amyloid (1‑42)‑induced memory impairment in mice. *Mol. Med. Rep*., 20(4): 3448–3455. [doi:10.3892/mmr.2019.10582](https://doi.org/10.3892/mmr.2019.10582).

Patil, S. P., Liu, C., Alban, J., Yang, N., and Li, X., 2014. Glycyrrhiza uralensis flavonoids inhibit brain microglial cell TNF-α secretion, p-IκB expression, and increase brain-derived neurotropic factor (BDNF) secretion. *Journal of Traditional Chinese Medical Sciences* 1(1): 28-37. doi:10.1016/j.jtcms.2014.11.004.

Hu, W. X., Xiang, Q., Wen, Z., He, D., Wu, X. M., and Hu, G. Z., 2014. Neuroprotective effect of Atractylodes macrocephalaon polysaccharides in vitro on neuronal apoptosis induced by hypoxia. *Mole. Med. Rep*. 9(6): 2573–2581. doi:10.3892/mmr.2014.2105.

Wang, N., Wang, X., He, M., Zheng, W., Qi, D., Zhang, Y., and Han, C. C., 2021. Ginseng polysaccharides: A potential neuroprotective agent. *J. Ginseng Res.* *45*(2), 211–217. [doi:10.1016/j.jgr.2020.09.002](https://doi.org/10.1016/j.jgr.2020.09.002).

Wattanathorn, J., Jittiwat, J., Tongun, T., Muchimapura, S., and Ingkaninan, K., 2011. Zingiber officinale Mitigates Brain Damage and Improves Memory Impairment in Focal Cerebral Ischemic Rat. *Evid. Based Complement. Alternat. Medi.: eCAM*, 429505. doi: 10.1155/2011/429505.

Wei, G., Da, H., Zhang, K., Zhang, J., Fang, J. and Yang, Z., 2021. Glycoside Compounds From Glycyrrhiza uralensis and Their Neuroprotective Activities. Nat. Prod. Commun. 16(2). doi:10.1177/1934578X21992988

Yang, E. J., Min, J. S., Ku, H. Y., Choi, H. S., Park, M. K., Kim, M. K., Song, K. S., and Lee, D. S., 2012. Isoliquiritigenin isolated from Glycyrrhiza uralensis protects neuronal cells against glutamate-induced mitochondrial dysfunction. *Biochem. Biophys. Res. Commun.* 421(4): 658–664. doi:10.1016/j.bbrc.2012.04.053.

Yang, Z., Zhang, D., Ren, J. et al., 2012. Acetylcholinesterase inhibitory activity of the total alkaloid from traditional Chinese herbal medicine for treating Alzheimer’s disease. *Med. Chem. Res.* 21: 734–738. doi:10.1007/s00044-011-9582-8

Ye, Y., Li, J., Cao, X., Chen, Y., Ye, C., and Chen, K., 2016. Protective effect of n-butyl alcohol extracts from Rhizoma Pinelliae Pedatisectae against cerebral ischemia-reperfusion injury in rats. *J.* *Ethnopharmacol.* 188: 259–265. [doi:10.1016/j.jep.2016.04.046](https://doi.org/10.1016/j.jep.2016.04.046).

Zhang, N., Liu, C., Sun, T. M., Ran, X. K., Kang, T. G., and Dou, D. Q., 2017. Two new compounds from Atractylodes macrocephala with neuroprotective activity. J. Asian Nat. Prod. Res. 19(1): 35–41. [doi:10.1080/10286020.2016.1247351](https://doi.org/10.1080/10286020.2016.1247351).

Zhou, X., Zhang, Y., Jiang, Y., Zhou, C., and Ling, Y., 2021. Poria cocos polysaccharide attenuates damage of nervus in Alzheimer's disease rat model induced by D-galactose and aluminum trichloride. *Neuroreport* 32(8): 727–737. [doi:10.1097/WNR.0000000000001648](https://doi.org/10.1097/WNR.0000000000001648).
